# Illinois State Medical Convention

**Published:** 1850-05

**Authors:** 


### ARTICLE II.

### ILLINOIS STATE MEDICAL CONVENTION.

We are gratified to hear, from the members of our profession in different parts of the state, that the prospects are good for a large, and general meeting of the physicians of Illinois, at Springfield, on the first Tuesday of June next. From Chicago, quite a number will go. And why not have a regular mass meeting of the profession? The objects are good, and the plan we doubt not meets the hearty approval of every intelligent and devoted member of the profession. The season of the year is that at which the health of our country is generally the best. The travelling facilities are then as good as at any other period; and the weather is usually pleasant. Under all these favorable circumstances, let each of our readers in Illinois, take the matter home to himself, and see if there be any good reason why he should not himself go, for all are privileged, and solicited to attend.—Can he not afford to spend the time once in his life, to meet in council, and friendly communion, his professional brethren? Can he not see in the addition it would make to his means of enjoyment, and the improvement both to his head and heart, that would almost inevitably result, that he would be greatly the gainer by going?

But we put the thing upon the higher ground of duty. Every physician that has the good of his fellow man at heart, is under obligations strong and binding, if in his power, to attend. For by the influence of association, we may very materially improve in our knowledge of diseases, and the means of curing them. Certainly annual reports from the able and talented members of our profession, in different parts of the state, can but exert this influence. And in proportion as we improve the means of curing disease, we elevate the standard of our profession. This is the key to medical reform. He who points out the means of curing one disease not before understood, does more to elevate the profession, than the enactments of an hundred Legislatures. for our protection. If community don't want protection against quacks, surely the profession does not. And those who use the means of improvement, by which they learn how more successfully to combat disease, are exerting more powerful influences to promote the good standing of our calling and to suppress quackery, than all they who write homilies upon professional dignity, or vend their sarcasm against empiricism.

Duty: yes, it is a duty to promote the improvemeut of all, by collecting together the experience, improvements, and wisdom of the various observing men in our ranks, by which we may all be enlightened, especially in reference to our endemic diseases, and hence become the better qualified to discharge the high and responsible duty of relieving the woes and infirmities of our fellow men. Pleasurc, interest, and duty, each emphatically, say to all who can, go to the convention, and lend your influence to carry out its benevolent designs.

Profs. Branard, Blaney and Herrick have been appointed delegates from Rush Medical College. E.

